# Examining paradoxical session attendance and weight loss relationships in a clinic based lifestyle modification intervention

**DOI:** 10.1002/osp4.696

**Published:** 2023-07-13

**Authors:** Kristen M. J. Azar, Sylvia Sudat, Qiwen Huang, Alice P. Pressman, Nina K. Szwerinski, Catherine Nasrallah, Elizabeth M. Venditti, Robert J. Romanelli

**Affiliations:** ^1^ Sutter Health Center for Health Systems Research Palo Alto California USA; ^2^ Department of Psychiatry & Department of Epidemiology Diabetes Prevention Support Center University of Pittsburgh Pittsburgh Pennsylvania USA

**Keywords:** diabetes mellitus, health education, lifestyle change, obesity, program effectiveness, type 2 diabetes

## Abstract

**Objective:**

Evaluations of lifestyle modification interventions (LMIs), modeled after the Diabetes Prevention Program, have repeatedly shown a dose‐response relationship between session attendance and weight loss. Despite this, not all participants had “average” weight loss experiences. Nearly one‐third of LMI participants experienced unexpected, paradoxical outcomes (i.e., high attendance with little weight loss, and low attendance with clinically significant weight loss). Paradoxical weight‐loss outcomes were characterized based on session attendance among participants in a group‐based LMI in a real‐world healthcare setting. This group‐based LMI was delivered over 1 year to participants with the possibility of attending up to 25 sessions total.

**Methods:**

LMI participants identified in 2010–2017 from electronic health records were characterized as having low (<75%) or high (≥75%) session attendance. Weight‐loss outcomes were defined as expected (≥5%, high‐attendance; <5%, low‐attendance) or paradoxical (≥5%, low‐attendance; <5%, high‐attendance). Paradoxical‐outcome‐associated characteristics were identified using logistic regression.

**Results:**

Among 1813 LMI participants, 1498 (82.6%) had low and 315 (17.4%) high session attendance; 555 (30.6%) had paradoxical outcomes, comprising 415 (74.8%) responders (≥5% weight‐loss) and 140 (25.2%) non‐responders (<5% weight‐loss). Among participants with high session attendance, paradoxical non‐responders were more likely to be female (odds ratio [OR]: 2.76; 95% confidence interval [CI]: 1.32, 5.77) and have type 2 diabetes (OR: 3.32; 95% CI: 1.01, 10.95). Among low‐attendance participants, paradoxical responders were more likely to be non‐Hispanic White and less likely to be non‐Hispanic Black (OR: 0.35; 95% CI: 0.18, 0.69), non‐Hispanic Asian (OR: 0.40; 95% CI: 0.22, 0.73), or Hispanic (OR: 0.53; 95% CI: 0.35, 0.80).

**Conclusions:**

In a healthcare setting, nearly one‐third of LMI participants experienced paradoxical outcomes. More research is needed to understand the facilitators and barriers to weight loss above and beyond session attendance.

## Graphical abstract

In a real‐world healthcare setting, participants in a group‐based lifestyle change program (LCP) were identified in 2010–2017 from electronic health records and characterized as having low (<75%) or high (≥75%) session attendance. Weight‐loss outcomes were defined as expected (≥5%, high‐attendance; <5%, low‐attendance) or paradoxical (≥5%, low‐attendance; <5%, high‐attendance). Nearly one‐third of LCP participants experienced paradoxical outcomes and subgroup differences were observed.

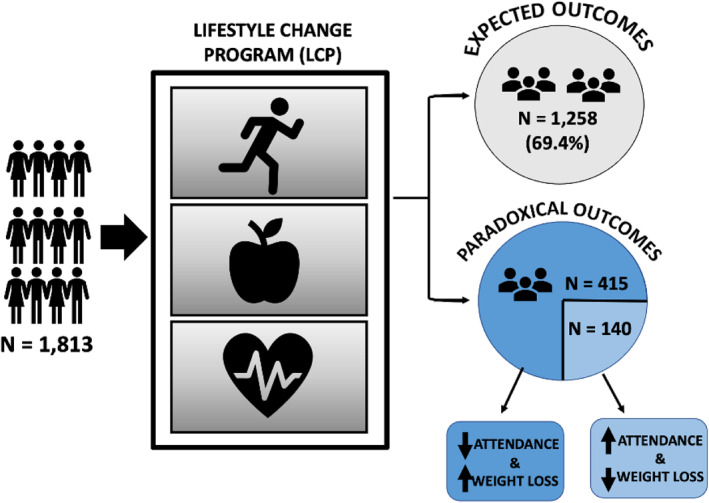

## 1 INTRODUCTION

In 2010, the U.S. Congress authorized the Centers for Disease Control (CDC) to enact the National Diabetes Prevention Program (NDPP), a collaboration of private and public organizations from across the nation promoting the dissemination of lifestyle modification interventions (LMIs) to achieve moderate sustainable weight loss, reduce the risk of developing type 2 diabetes, and improve overall health.^1^ CDC‐aligned LMIs were modeled after the efficacious Diabetes Prevention Program (DPP),^2^ an intervention tested via a randomized controlled trial (RCT) of an intensive individually administered LMI focused on the reduction of overall calorie and fat intake as well as adoption of moderate regular physical activity. The DPP trial resulted in an average weight loss of 7% and decreased the risk of onset of type 2 diabetes by 58% during 2.8 years of follow‐up in adults with impaired glucose tolerance compared with a placebo control group.^3^ In contrast, metformin decreased the risk of type 2 diabetes by 31% and was found to be significantly inferior to the LMI.^4^

Studies of CDC‐aligned translational interventions,^5^, ^6^, ^7^, ^8^, ^9^ which deliver year‐long group‐based LMIs of 16 sessions in the first 6 months followed by roughly once‐per‐month maintenance sessions, have shown a dose‐response relationship between session attendance and weight loss. Retention in these programs is a persistent issue; for example, Ely and colleagues^6^ found that among group‐based NDPP LMI participants across multiple cohorts of more than 14,000 middle‐aged adults, only 43% completed all 16 sessions of the program compared to 95% from the original DPP trial.^10^ Nevertheless, session attendance was positively associated with weight loss, with each NDPP session attended corresponding on average to a 0.31% reduction in body weight (or approximately 5% loss of body weight over the course of 16 sessions).^6^

Evaluations from LMIs, modeled after the DPP, have repeatedly shown a dose‐response relationship between session attendance and weight loss. Despite a dose‐response relationship at the population level, weight loss is a heterogeneous phenomenon at the subgroup or the individual level where not all participants have “average” weight loss experiences. Some individuals experience unexpected, seemingly paradoxical outcomes (i.e., high attendance with little weight loss, and low attendance with clinically significant weight loss). Multiple studies have characterized heterogeneous weight changes among participants in CDC‐aligned LMIs.^11^, ^12^, ^13^ For example, our prior work showed that on average, group‐based LMI participants lost 3.7% (95% CI: −3.9%, −3.5%) body weight at 12‐week follow‐up, but clustering analyses revealed three distinct patterns of weight loss.^14^ These were characterized as minimal‐to‐no weight loss, delayed‐minimal weight loss, and steady moderate weight loss, equivalent to mean weight changes of 0.4%, −2.3%, and −4.8% at a 12‐week follow‐up, respectively.^14^ Notably, these short‐term changes in weight were highly correlated with weight loss outcomes at the completion of the program (52‐week follow‐up) and were dependent upon levels of participation. However, despite the average direct relationship between weight loss and session attendance, not all participants had “average” weight loss experiences. In fact, some with low attendance achieved program‐defined weight loss goals, whereas others with high attendance did not. Understanding these unexpected and paradoxical outcomes is important, especially for participants who attend most (or all) sessions but do not achieve clinically meaningful weight loss (defined as 5% of body weight).^15^ To our knowledge, there are no studies that have examined these paradoxical outcomes.

Using data from electronic health records (EHR) of a large integrated healthcare system in Northern California, the authors sought to examine session attendance and weight‐loss outcomes of participants in a CDC‐aligned group‐based LMI to identify and characterize patterns of paradoxical outcomes. The hypothesis was that such outcomes could be associated with differences in comorbidity burden and overall metabolic risk at baseline.

## 2 METHODS

A longitudinal descriptive analysis of LMI‐enrolled participants^9^ using EHR data from an integrated healthcare delivery system serving a geographically diverse patient population in northern California, United States (2010–2017) was conducted. Details of the lifestyle modification program are described elsewhere (Huang et al.^16^).

Patients were categorized into four mutually exclusive groups based on session attendance and weight loss goal achievement. Specifically, participants were characterized as having low (<75% of sessions completed) or high (≥75% sessions completed) attendance, and as LMI responders (≥5% weight loss) or non‐responders (<5%) from baseline to 12 months follow‐up. The 75% cut‐point used in our analysis was derived from the assertion that 19 out of 25 sessions are considered a “good” attendance or proxy of engaged program participation. This cut‐point has been used in other studies.^17^ This study was approved by the Sutter Health Institutional Review Board.

### 2.1 Cohort identification

LMI participants were identified as having at least one encounter for the program recorded in the EHR between 2010 and 2017. The first LMI encounter was considered the index date. Eligible cohort patients were required to be at least 18 years of age (as of the index date) and to have EHR activity during the 12–36 months prior to the index date to confirm previous contact with the system and to gather medical history. Patients were also required to have a baseline weight measurement captured on or up to 12 months prior to the index date and a follow‐up weight measurement captured at 12 months (±3 months) from the index date. All participants included in the analysis enrolled in the full 12‐month program with the possibility of attending up to 25 sessions total over the year.

Exclusion criteria included patients diagnosed with International Classification of Disease (ICD) 9 or 10 indicating potential dramatic weight change due to pregnancy, metastatic cancer, gastric bypass surgery, chronic liver disease, or end‐stage kidney disease in the 12 months prior or up to 24 months after index date (tab. A1 in Romanelli et al.^14^).

### 2.2 Covariates

Baseline demographics, clinical characteristics, and healthcare utilization were extracted from the EHR in the 12 months prior to the index date. Demographic variables included age, primary insurance, self‐reported gender, race/ethnicity, and preferred spoken language. Race and ethnicity were defined by Hispanic ethnicity, followed by racial group. If a patient did not self‐identify as Hispanic, they were classified based on their self‐reported race. Throughout this article, references to racial groups imply non‐Hispanic. This paper used the designation “Hispanic” instead of the commonly used term “Latinx” for consistency with US Census categories^18^ and self‐reported data collection. Based on program participants' available home addresses, median income by zip code was used as a proxy for socioeconomic status. Baseline clinical characteristics including weight, body mass index (BMI), smoking status, and active prescriptions. Co‐morbidities were identified based on a combination of ICD 9/10 codes, medication orders, and/or laboratory values in EHR, including type 2 diabetes, prediabetes/increased risk of type 2 diabetes, metabolic syndrome, hypertension, dyslipidemia, atherosclerotic cardiovascular disease, depression, and anxiety. The Charlson co‐morbidity index (CCI) was also calculated.^19^ The CCI score^20^ has been validated and reported to have a strong ability to predict mortality.^21^ The higher the score, the higher likely the severity of comorbidity. The CCI score was calculated using the diagnostic code from the problem list, encounters, and billing data. Healthcare utilization included a number of outpatient visits and telephone/electronic visits, preventive visits, and documented influenza immunization.

### 2.3 Outcomes

The outcomes of interest were the occurrence of a paradoxical (i.e., unexpected) outcome (Yes/No) defined as **paradoxical responder**: low session attendance (<75% sessions) and significant weight loss (≥5%); or **paradoxical non‐responder**: high session attendance (≥75% sessions) and insignificant weight loss (<5%).^22^ Conversely, non‐paradoxical (i.e., expected) outcomes were defined as **expected responder**: high session attendance and significant weight loss; or **expected non‐responders**: low session attendance with low weight loss.

### 2.4 Statistical analyses

The authors summarized quasi‐continuous variables with means and standard deviations (SD) and categorical variables with counts and proportions for the four mutually exclusive groups: (1) paradoxical responders; (2) paradoxical non‐responders; (3) expected responders; and (4) expected non‐responders. Comparisons were made between paradoxical response versus expected response for low session attendance and paradoxical non‐response versus expected response for high session attendance, respectively. Chi‐square tests and Fisher's exact tests were used for categorical variables and *t*‐tests for quasi‐continuous variables.

Within the low‐attendance (Model 1) and high‐attendance (Model 2) groups, logistic regression was used to assess the probability of paradoxical versus an expected weight‐loss outcome adjusting for patient demographics and clinical characteristics. Adjusted odds ratios (OR) and 95% confidence intervals (CI) were calculated from both logistic regression models. All statistical analysis was conducted in SAS v9.4 (SAS Institute).

## 3 RESULTS

A total of 1813 participants met the eligibility criteria. For participants, 94.2% had a baseline weight that was measured on the date of the first LMI visit. Furthermore, 99.7% of participants had a baseline weight measured within 3 months of the initial LMI visit. Overall, 590 (32.5%) participants attained ≥5% weight loss. The majority of participants (82.6%, 1498) had low session attendance. A total of 1083 participants (86.0%) were classified as expected non‐responders (low session attendance, <5% weight loss) and 175 (14.0%) as expected responders (high session attendance, ≥5% weight loss). The remaining 555 (30.6%) participants had unexpected outcomes; 415 (74.8%) were classified as paradoxical responders (low session attendance, ≥5% weight loss) and 140 (25.2%) were classified as paradoxical non‐responders (high session attendance, <5% weight loss).

Demographic, clinical, and healthcare utilization characteristics of participants with expected and paradoxical (unexpected) outcomes are shown in Table 1. Among participants with low session attendance, paradoxical responders (i.e., those who unexpectedly lost weight despite lower attendance) were slightly older (mean age 54.4 vs. 52.5 years, *p* = 0.01). The racial/ethnic composition of these participants differed; those self‐identifying as non‐Hispanic White were twice as likely to experience a paradoxical response as compared to non‐Hispanic Black or non‐Hispanic Asian participants (32% vs. 16%, *p* < 0.001). Paradoxical responders also tended to have a higher CCI score than expected non‐responders, with 8.9% versus 5.4% scoring a CCI of 3 or more, respectively (*p* = 0.04).

**TABLE 1:** Demographics and clinical characteristics by session attendance and expected versus paradoxical outcomes.

	Low session attendance (<75% sessions)		*p*‐Value	High session attendance (≥75% sessions)		*p*‐Value
	Paradoxical response (≥5% weight loss)	Expected response (<5% weight loss)		Paradoxical non‐response (<5% weight loss)	Expected response (≥5% weight loss)	
	*N* = 415	*N* = 1083		*N* = 140	*N* = 175	
Demographics						
Age	54.4 ± 13.0	52.5 ± 13.2	0.01	56.8 ± 11.8	57.0 ± 11.2	0.88
Female, *n* (%)	321 (77.3)	833 (76.9)	0.86	117 (83.6)	128 (73.1)	0.03
Race/ethnicity, *n* (%)			<0.001			0.60^a^
Non‐Hispanic Black	11 (2.7)	58 (5.4)		3 (2.1)	6 (3.4)	
Non‐Hispanic Asian	15 (3.6)	79 (7.3)		7 (5.0)	5 (2.9)	
Hispanic	34 (8.2)	141 (13)		6 (4.3)	14 (8.0)	
Non‐Hispanic White	325 (78.3)	700 (64.6)		110 (78.6)	131 (74.9)	
Non‐Hispanic other	4 (1)	19 (1.8)		1 (0.7)	3 (1.7)	
Unknown	26 (6.3)	86 (7.9)		13 (9.3)	16 (9.1)	
Insurance payer, *n* (%)			0.24			0.50^a^
Commercial FFS/PPO/HMO	247 (59.5)	685 (63.3)		94 (67.1)	108 (61.7)	
Commercial FFS/PPO	177 (42.7)	499 (46.1)		64 (45.7)	73 (41.7)	
Commercial HMO	70 (16.9)	186 (17.2)		30 (21.4)	35 (20)	
Medicare (FFS/HMO)/Medicaid	94 (22.7)	202 (18.7)		36 (25.7)	46 (26.3)	
Medicare (FFS/HMO)	84 (20.2)	184 (17)		35 (25)	43 (24.6)	
Medicaid	10 (2.4)	18 (1.7)		1 (0.7)	3 (1.7)	
Other/self	7 (1.7)	28 (2.6)		2 (1.4)	6 (3.4)	
Unknown	67 (16.1)	168 (15.5)		8 (5.7)	15 (8.6)	
English proficient, *n* (%)	406 (97.8)	1054 (97.3)	0.57	138 (98.6)	172 (98.3)	1.0^a^
Median household income, *n* (%)			0.83			0.77
<$50,000	69 (16.6)	170 (15.7)		16 (11.4)	19 (10.9)	
≥$50,000–<$75,000	116 (28)	317 (29.3)		34 (24.3)	48 (27.4)	
≥$75,000–<$100,000	96 (23.1)	262 (24.2)		39 (27.9)	47 (26.9)	
≥$100,000	105 (25.3)	249 (23)		41 (29.3)	54 (30.9)	
Unknown income	29 (7)	85 (7.8)		10 (7.1)	7 (4)	
Mean number of sessions attended,^d^ *n* (%)	10.1 (40.4)	8.3 (33.3)		20.8 (83.3)	21.0 (84.1)	
Clinical characteristics						
Mean weight, kg ± SD	101.2 ± 22.2	101.1 ± 23.6	0.92	101.8 ± 25.4	101.3 ± 23.6	0.86
BMI,^e^ kg/m^2^	36.2 ± 6.8	36.3 ± 7.0	0.69^b^	36.8 ± 7.1	35.9 ± 7.0	0.25^b^
BMI category,^f^ kg/m^2^			0.60^ac^			0.60^ac^
<25	8 (1.9)	24 (2.2)		4 (2.9)	4 (2.3)	
25–<30	73 (17.6)	164 (15.1)		15 (10.7)	26 (14.9)	
30+	334 (80.5)	893 (82.5)		121 (86.4)	144 (82.3)	
Missing	0 (0)	2 (0.2)		0 (0)	1 (0.6)	
Smoking status, *n* (%)			0.55			0.75^a^
Current	28 (6.7)	65 (6)		2 (1.4)	5 (2.9)	
Ever	123 (29.6)	288 (26.6)		38 (27.1)	42 (24)	
Never	260 (62.7)	716 (66.1)		98 (70)	124 (70.9)	
Unknown	4 (1)	14 (1.3)		2 (1.4)	4 (2.3)	
Established PCP, *n* (%)	382 (92.0)	1014 (93.6)	0.28	132 (94.3)	162 (92.6)	0.54
Risk group, *n* (%)			0.19			0.001^a^
Type 2 diabetes	103 (24.8)	294 (27.1)		48 (34.3)	30 (17.1)	
Prediabetes/high risk diabetes	210 (50.6)	481 (44.4)		63 (45)	103 (58.9)	
Overweight/obesity with no metabolic condition	96 (23.1)	291 (26.9)		24 (17.1)	40 (22.9)	
Other risk	6 (1.4)	17 (1.6)		5 (3.6)	2 (1.1)	
Clinical conditions						
Metabolic syndrome, *n* (%)	115 (27.7)	302 (27.9)	0.95	44 (31.4)	33 (18.9)	0.01
Hypertension, *n* (%)	204 (49.2)	516 (47.6)	0.60	63 (45)	73 (41.7)	0.56
Dyslipidemia, *n* (%)	205 (49.4)	472 (43.6)	0.04	79 (56.4)	87 (49.7)	0.24
ASCVD, *n* (%)	34 (8.2)	79 (7.3)	0.56	10 (7.1)	11 (6.3)	0.76
Depression, *n* (%)	96 (23.1)	282 (26.0)	0.25	31 (22.1)	33 (18.9)	0.47
Anxiety	41 (9.9)	99 (9.1)	0.66	12 (8.6)	14 (8)	0.85
Charlson comorbidity score (CCI score)			0.04			0.0006
0	213 (51.3)	559 (51.6)		64 (45.7)	112 (64)	
1–2	165 (39.8)	465 (42.9)		64 (45.7)	60 (34.3)	
3+	37 (8.9)	59 (5.4)		12 (8.6)	3 (1.7)	
Prescriptions						
Mean active prescriptions, count ± SD	4.8 ± 3.8	4.7 ± 3.8	0.74^b^	4.7 ± 3.7	3.6 ± 3.1	0.003^b^
			0.93^c^			0.03^c^
0	35 (8.4)	107 (9.9)		14 (10)	23 (13.1)	
1–2	98 (23.6)	254 (23.5)		26 (18.6)	54 (30.9)	
3–4	93 (22.4)	242 (22.3)		40 (28.6)	40 (22.9)	
5–6	76 (18.3)	198 (18.3)		23 (16.4)	30 (17.1)	
>6	113 (27.2)	282 (26)		37 (26.4)	28 (16)	
Weight loss drug, *n* (%)	31 (7.5)	88 (8.1)	0.67	11 (7.9)	9 (5.1)	0.33
Weight loss diabetes drug, *n* (%)	63 (15.2)	196 (18.1)	0.18	29 (20.7)	21 (12)	0.04
Other diabetes drugs, *n* (%)	37 (8.9)	118 (10.9)	0.26	22 (15.7)	10 (5.7)	0.004
Baseline healthcare utilization^g^						
Number of outpatient ambulatory visits, mean ± SD	9.7 ± 9.2	9.0 ± 8.7	0.21	11.4 ± 15.1	8.3 ± 8.8	0.03
Number of telephone/electronic visits, mean ± SD	14.1 ± 12.8	14.3 ± 13.9	0.85	14.1 ± 11.6	11.9 ± 10.1	0.07
Preventive visit, *n* (%)	156 (37.6)	362 (33.4)	0.13	54 (38.6)	80 (45.7)	0.21
Influenza immunization, *n* (%)	95 (22.9)	250 (23.1)	0.94	61 (43.6)	55 (31.4)	0.03

Abbreviations: ASCVD, atherosclerotic cardiovascular disease; BMI, body mass index; FFS, fee‐forservice; HMO, health maintenance organization; PCP, primary care provider; PPO, preferred provider organization.

^a^ Fisher's Exact test.

^b^ For continuous variable.

^c^ For categorical variable.

^d^ Total number of sessions possible is 25.

^e^ *n* = 3 missing BMI.

^f^ Categories differ for those self‐identifying as non‐Hispanic Asian: <23, 23–<27, 27+.

^g^ The baseline healthcare utilization variables were in 1 year prior to the index date. These visits were non‐LMI visits. Preventive visits were defined by the CPT codes '9938', '9939', 'G0344', 'G0402', 'G0438', and 'G0439' using billing data. Influenza immunization was defined by a procedure code from the billing data. The number of outpatient visits and number of telephone/electronic visits were defined from patient encounter data.

Among participants with high session attendance, paradoxical non‐responders (i.e., those who unexpectedly did not achieve 5% weight loss despite higher attendance) were less frequently male than expected responders (16.4% vs. 26.9%, *p* = 0.03); no differences were observed in age or race/ethnicity. Paradoxical non‐responders had higher CCI scores than expected responders (54.3% vs. 36% with CCI 1+, *p* < 0.001) and were more likely to have type 2 diabetes (34.3% vs. 17.1%, *p* = 0.001), metabolic syndrome (31.4% vs. 18.9%, *p* = 0.01), and to take weight‐loss‐promoting diabetes medication (20.7% vs. 12%, *p* = 0.04) or other diabetes medication(s) (15.7% vs. 5.7%, *p* = 0.004). Additionally, paradoxical non‐responders had higher ambulatory healthcare utilization in the prior year as compared to the expected responders, including more ambulatory visits on average (11.4 vs. 8.3 visits per participant, *p* = 0.03), and were more likely to have recorded influenza vaccinations (43.6% vs. 31.4%, *p* = 0.03).

Among those with low attendance, after adjusting for other covariates (Table 2, Model 1), a CCI score of 3 or more was associated with nearly twice the odds of being a paradoxical responder versus an expected non‐responder (OR: 1.99; 95% CI: 1.13, 3.52). Similar to the unadjusted analysis, paradoxical responders were also more likely to be non‐Hispanic White and less likely to be non‐Hispanic Black (OR: 0.35; 95% CI: 0.18, 0.69), non‐Hispanic Asian (OR: 0.40; 95% CI: 0.22, 0.73), or Hispanic (OR: 0.53; 95% CI: 0.35, 0.80). Among participants with high attendance, females had 2.76 (95% CI: 1.32, 5.77) times the odds of being a paradoxical non‐responder than an expected responder (Table 2, Model 2); those with diabetes had three times the odds of not responding as compared to those with no metabolic conditions (OR: 3.33; 95% CI: 1.01, 10.9).

**TABLE 2:** Odds of paradoxical outcomes from logistic model adjusted by patient demographics and clinical characteristics.

	Model 1: Low attendance cohort: Odds of being a paradoxical responder versus an expected non‐responder^a^	Model 2: High attendance cohort: Odds of being a paradoxical non‐responder versus an expected responder^b^
	Adjusted odds ratio (95% CI)	Adjusted odds ratio (95% CI)
Demographics		
Age	1.00 (0.99, 1.01)	0.99 (0.96, 1.02)
Female	1.09 (0.79, 1.50)	2.76 (1.32, 5.77)
Race/ethnicity		
Non‐Hispanic White	Reference	Reference
Non‐Hispanic Black	0.35 (0.18, 0.69)	0.52 (0.10, 2.86)
Non‐Hispanic Asian	0.40 (0.22, 0.73)	1.53 (0.37, 6.41)
Hispanic	0.53 (0.35, 0.80)	0.43 (0.14, 1.35)
Non‐Hispanic other	0.46 (0.15, 1.40)	0.35 (0.03, 3.86)
Unknown	0.61 (0.37, 1.00)	1.30 (0.50, 3.38)
Insurance payer		
Commercial FFS/PPO/HMO	Reference	Reference
Medicare (FFS/HMO)/Medicaid	1.08 (0.76, 1.54)	0.71 (0.34, 1.48)
Other/self	0.60 (0.25, 1.41)	0.46 (0.07, 3.08)
Unknown	1.18 (0.82, 1.70)	0.41 (0.13, 1.24)
English proficient	0.85 (0.38, 1.94)	1.77 (0.17, 18.95)
Median household income		
<$50,000	Reference	Reference
≥$50,000–<$75,000	0.88 (0.61, 1.27)	0.89 (0.34, 2.32)
≥$75,000–<$100,000	0.89 (0.61, 1.30)	1.22 (0.47, 3.17)
≥$100,000	1.02 (0.69, 1.49)	1.13 (0.43, 2.94)
Unknown income	0.87 (0.51, 1.47)	1.19 (0.28, 4.96)
Clinical characteristics		
Mean weight, kg ± SD	1.00 (0.99, 1.01)	1.00 (0.99, 1.02)
Smoking status		
Current	Reference	Reference
Ever	0.76 (0.45, 1.28)	1.9 (0.28, 12.65)
Never	0.71 (0.43, 1.15)	1.94 (0.31, 12.06)
Unknown	0.44 (0.13, 1.54)	1.63 (0.09, 30.68)
Established PCP	0.72 (0.46, 1.15)	1.30 (0.38, 4.44)
Risk group		
Overweight/obesity with no metabolic condition	Reference	Reference
Prediabetes/high risk diabetes	1.22 (0.86, 1.73)	1.61 (0.75, 3.48)
Type 2 diabetes	1.00 (0.58, 1.75)	3.32 (1.01, 10.95)
Other risk	1.13 (0.41, 3.09)	2.28 (0.24, 22.02)
Clinical conditions		
Metabolic syndrome	0.99 (0.69, 1.42)	1.58 (0.69, 3.63)
Hypertension	0.92 (0.68, 1.24)	0.66 (0.33, 1.32)
Dyslipidemia	1.24 (0.94, 1.63)	1.06 (0.59, 1.92)
ASCVD	0.92 (0.58, 1.45)	0.75 (0.25, 2.26)
Depression	0.76 (0.56, 1.03)	0.97 (0.46, 2.03)
Anxiety	1.12 (0.74, 1.69)	0.65 (0.23, 1.81)
CCI score		
0	Reference	Reference
1–2	1.06 (0.78, 1.43)	1.18 (0.61, 2.27)
3+	1.99 (1.13, 3.52)	4.05 (0.71, 23.24)
Prescriptions		
0	Reference	Reference
1–2	1.11 (0.70, 1.78)	0.86 (0.35, 2.15)
3–4	1.07 (0.65, 1.76)	1.25 (0.47, 3.34)
5–6	1.12 (0.66, 1.89)	0.88 (0.29, 2.66)
>6	1.09 (0.62, 1.91)	0.83 (0.24, 2.88)
Types of medications		
Weight loss drug	0.96 (0.60, 1.53)	1.81 (0.60, 5.47)
Weight loss diabetes drug	0.94 (0.60, 1.45)	0.85 (0.30, 2.38)
Other diabetes drugs	0.78 (0.47, 1.3)	1.56 (0.44, 5.50)
Healthcare utilization		
Number of outpatient ambulatory visits	1.02 (1.00, 1.04)	1.03 (1.00, 1.06)
Number of telephone/electronic visits	0.99 (0.98, 1.00)	0.99 (0.96, 1.03)
Preventive visit	1.3 (0.99, 1.70)	0.59 (0.34, 1.04)
Influenza immunization	0.96 (0.71, 1.29)	1.73 (0.98, 3.06)

Abbreviations: ASCVD, atherosclerotic cardiovascular disease; FFS, fee‐forservice; HMO, health maintenance organization; PCP, primary care provider; PPO, preferred provider organization.

^a^ Model 1: Odds of response (≥5% weight loss; *n* = 415) versus expected outcome (<5% weight loss; *n* = 1083) among patients who attended less than 75% classes at 12 months follow‐up.

^b^ Model 2: Odds of non‐response (<5% weight loss; *n* = 140) versus expected outcome (≥5% weight loss; *n* = 175) among patients who attended 75% or more classes at 12 months follow‐up.

## 4 DISCUSSION

In this study, the authors sought to examine paradoxical or unexpected outcomes from a CDC‐aligned group‐based LMI in a large integrated healthcare system in Northern California. Given the concerns and efforts to address low engagement and long‐term retention for these types of LMIs, it is worthwhile to characterize paradoxical responders and non‐responders.^8^, ^23^ Strikingly, it was found that only 17.4% of participants attended more than 75% of the sessions offered. Yet, 32.5% achieved ≥5% weight loss and sustained it at 12 months from baseline.^9^

Characteristics associated with both paradoxical *response* (i.e., low session attendance and ≥5% body weight loss) and *non‐response* (i.e., high session attendance and <5% body weight loss) were explored. Paradoxical outcomes occurred for nearly one‐third of the cohort (30.6%), of which 415 (74.8%) were responders (with low session attendance) and 140 (25.2%) were non‐responders (with high session attendance). The rates of paradoxical outcomes within each attendance group differed; 27.7% of those in the low‐attendance cohort experienced paradoxical outcomes, as compared to 44.4% of those in the high‐attendance cohort. These findings underscore that the “dose‐response” relationship between session attendance and weight loss is an average effect and that not all participants experience “average” outcomes; rather, weight loss is a heterogenous phenomenon.

The authors initially hypothesized that participants with high session attendance and low weight loss were more likely to have co‐morbid conditions and higher overall metabolic risk at baseline. These patients with type 2 diabetes were found to be three times more likely to be non‐responders than those without metabolic conditions. The LMI was designed for patients with pre‐diabetes among those with higher glucose levels, and therefore may not have provided sufficient support for those already diagnosed with type 2 diabetes. Self‐management, including diabetes self‐management education, may need to be more multifaceted for those with the greatest cardiometabolic burden.

Females within the high‐attendance cohort had nearly 3‐fold odds of non‐response compared with males, after adjusting for other sociodemographic, clinical, and healthcare utilization factors. Several studies^12^, ^24^, ^25^ have found that males lose more weight than females in weight loss interventions. It is beyond the scope of this study whether paradoxical non‐responders had lower levels of self‐efficacy or other social drivers of health and other lifestyle factors (e.g., occupation, family support structure, sleep habits, caregiver status) that can influence health behaviors. Furthermore, there could be undiagnosed hormonal imbalances, especially among women, or mental health factors. For example, hypothyroidism, the second most common endocrine disorder among women, has been found to be associated with weight gain and obesity.^26^ Depression and anxiety have been associated with both weight loss^27^, ^28^, ^29^ and weight gain^27^, ^28^, ^30^, ^31^, representing a paradox that has been much studied. In our study, diagnoses of depression or anxiety were not found to be associated with paradoxical outcomes (either paradoxical response or non‐response).

It is possible that genetic factors could contribute to paradoxical non‐response despite high session attendance. Genetic predisposition to human obesity risk is conditioned by thousands of DNA variants that make obesity prevention and management a major challenge.^32^ Several studies have shown that the heritability of a genetic variant for obesity and central fat distribution among individuals having endemic obesity is approximately 50% compared to 35% among individuals who have normal weight.^33^, ^34^, ^35^, ^36^ A study conducted by Frayling et al. (2007) showed that the intron 1, a common variant of the fat mass and obesity (FTO) gene, is associated with BMI and predisposes individuals to obesity.^37^ In addition, Scuteri et al. (2007), who performed a genome association scan to identify genetic variants associated with obesity, found that FTO genetic variants are associated with increased changes in the individuals' BMI, hip circumference, and body weight.^38^ These changes could have a significant effect on the risk of developing obesity. These contributors underscore an urgent need for the identification of better therapeutic targets and the development of effective medications. This also requires a shift in thinking from purely behavioral to biological barriers to weight loss that remain poorly understood.

Interestingly, in the low‐attendance cohort, a higher CCI score was associated with a paradoxical response in the fully adjusted model. This result is difficult to interpret without further investigation into the types of comorbidities and concurrent interventions that could have affected weight‐loss outcomes within this small subgroup (6% of the low‐attendance cohort). For example, given the potential need in this group for more rapid risk reduction, it is possible that this subgroup of participants was more likely to be engaged in additional external concurrent interventions. For this analysis, individuals who underwent bariatric surgery were excluded; however, this study did not have information about other weight loss programs, behavior change/health education courses, or other health or psychosocial interventions that participants could have engaged in.

The largest contributor to the paradoxical response in the low‐attendance cohort was non‐Hispanic White race/ethnicity. Even after adjustment for various other sociodemographic and clinical factors, non‐Hispanic White participants were almost twice as likely as Hispanic participants, 2.5 times as likely as non‐Hispanic Asian participants, and almost 3 times as likely as non‐Hispanic Black participants to experience positive weight‐loss outcomes despite low session attendance. Studies have previously documented lower success at weight‐loss goal achievement through LMIs for non‐Hispanic Black participants specifically, and there is increasing study regarding how socio‐cultural factors and longstanding societal inequities, including the daily stress of discrimination, could be contributing to worse health outcomes for people from historically marginalized communities.^39^, ^40^, ^41^, ^42^ However, our study contributes to the new finding that non‐Hispanic White participants are more likely to achieve weight loss goals despite low engagement, which could suggest that non‐Hispanic White patients experience better healthcare overall (access, racial concordance, quality of care) and societal privileges than racially and ethnically minoritized people. This underscores the need for both further research with Hispanics, non‐Hispanic Blacks, non‐Hispanic Asians, and individuals of other non‐Hispanic communities to better understand barriers and unmet needs in the context of LMIs, and also the development of tailored LMIs that are culturally sensitive and able to respond to those needs.^43^ This finding also highlights the need to include measures of racism and more detailed social and economic measures when evaluating LMIs.^44^, ^45^, ^46^, ^47^

It is important to note that the LMI examined was only offered in‐person and without a telehealth option to facilitate virtual attendance and/or remote participation. It is unclear how the acceleration of telehealth may impact engagement in LMIs for patients. Some evidence suggests that the effectiveness was lower among online diabetes prevention LMI participants compared to in‐person participants.^48^ Furthermore, more adults referred to online programs enrolled, but fewer engaged at 6‐month compared to in‐person participants.^48^ This may, in part, be due to a need to design programs intended for telehealth delivery as opposed to adapting a program that was designed for an in‐person setting. Additionally, it may be important to identify which patient segments and subgroups may benefit from this modality, given barriers as well as preferences. More work is needed to optimize this modality for maximum effectiveness.

This study has limitations that should be considered when interpreting the findings. First, study data were derived from an open healthcare system, meaning that individuals can receive care outside the system. Accordingly, some diagnoses or other covariates may have been missed; however, the program is restricted to patients who receive primary care at Sutter Health, and therefore are considered likely to receive most of their ambulatory care within the system. Second, because this study was retrospective and patient‐reported data were not collected, behavioral factors or other social factors that may impact program engagement could not be measured. Therefore, unmeasured confounding is likely. Despite these limitations, data were from a large health healthcare system and therefore our results have good generalizability to other health systems across the US.

## 5 CONCLUSION

Approximately one third of participants from a group‐based LMI in Northern California experienced paradoxical outcomes. Nearly half the individuals who attended at least 75% of program sessions did not achieve clinically significant weight loss (paradoxical non‐responders) and were more likely to be female and have type 2 diabetes after adjusting for other factors. In contrast, those with low attendance who achieved weight‐loss goal achievement (paradoxical responders) were more likely to self‐report as non‐Hispanic White and have higher CCI scores than those who did not. This study emphasizes that there are participants in LMIs whose weight loss patterns do not follow average trends and highlights the need for both more research to understand individual weight loss facilitators and barriers, and to develop more personalized, culturally tailored and informed weight loss strategies.

## AUTHOR CONTRIBUTIONS

All authors declare that they have no conflicts of interest or financial disclosures. All authors listed have met the International Committee of Medical Journal Editors (ICMJE) requirements for authorship and have made significant intellectual contributions to this work. Authors Azar and Romanelli designed the study. Authors Azar, Romanelli and Huang directed its implementation, including quality assurance and control. Authors Azar and Romanelli designed the statistical analyses, and author Huang conducted the analyses. All authors contributed to the literature review and discussion, and to the revision of the manuscript.

## CONFLICT OF INTEREST STATEMENT

The authors declare no conflicts of interest.
